# Correlates of suicidal ideation in rural Chinese junior high school left-behind children: A socioecological resilience framework

**DOI:** 10.3389/fpsyt.2022.901627

**Published:** 2022-07-22

**Authors:** Yu-ming Zhou, Leona Mak, Chun-xia Zhao, Fan He, Xiao-na Huang, Xiao-bo Tian, Yi-zheng, Jing Sun

**Affiliations:** ^1^Beijing Key Laboratory of Mental Disorders, The National Clinical Research Center for Mental Disorders, Beijing Institute for Brain Disorders, Beijing Anding Hospital, Capital Medical University, Beijing, China; ^2^Menzies Health Institute Queensland, School of Medicine and Dentistry, Griffith University, Gold Coast, QLD, Australia; ^3^United Nations Children's Fund (UNICEF China Office), Beijing, China

**Keywords:** suicide, left-behind children, resilience, emotional and behavioral problems, self-harm

## Abstract

**Introduction:**

Suicide is one of the top five causes of adolescent mortality around the world. The socioecological resilience framework in explaining the risk factors and protective factors for suicidal ideation in left-behind children (LBC) has not been well explored. The current study aims to compare the prevalence of suicidal ideation in LBC and non-LBC, and explore its correlations with resilience factors among LBC.

**Methodology:**

This study was part of an epidemiological survey conducted by UNICEF exploring mental health outcomes in left-behind children. We implemented a cross-sectional study collecting data from 11 provinces and 1 municipal, with 5,026 participants (3,359 LBC, 1,667 controls) in year one junior high school living in impoverished areas of rural China. Data on suicidal ideation, self-harm, resilience factors including health-risk behaviors, psychological wellbeing as it was measured by the Strengths and Difficulties Questionnaire, peer relationship within the school environment, and family support were collected.

**Results:**

Overall prevalence of suicidal ideation among LBC was 7.2% which is significantly different from 5.5% reported by NLBC (χ^2^ = 4.854, *p* = 0.028). LBC reported a higher prevalence of self-harm (16.4%) than NLBC (13.0%; χ^2^ = 10.232, *p* = 0.001), but there was no difference in the prevalence of suicide plan, suicide attempt or help-seeking. LBC had significantly poorer psychological feeling, and greater emotional and behavioral difficulties peer relationship in the school environment than controls. In the multiple logistic regression, history of self-harm was the greatest predictor for suicidal ideation among LBC (OR = 2.078, 95% CI: 1.394–3.100, *p* < 0.001). Health risk behavior including previous smoking attempt, poor psychological feeling, and emotional and behavior difficulties, and poor peer relationship within school environment, were also significant risk factors for suicidal ideation among LBC.

**Conclusion:**

The prevalence of suicidal ideation and self-harm was greater among left-behind than non-left-behind children. Our results show resilience factors including previous self-harm, emotional and behavioral problems, smoking, and poor peer relationship are significantly associated with suicidal ideation in left-behind adolescents.

## Introduction

Left-behind children are a unique group of children who have been subject to separation from one or both parents who have migrated for at least 6 months (1). Labor migration describes the migration of parents living in low-income areas to regions of higher income in search for better employment, opportunity, and lifestyle. Their children usually remain in the hometown and are cared for by their grandparents, other relatives, or the wider community. According to UNICEF China 2015 Reports, there are currently 69 million children who identify as being “left-behind” which estimates to every four out of ten Chinese children being directly affected by parental migration (2).

Childhood and adolescence are a crucial period in a person's life where an individual develops a sense of self and the world they live in (3). Parents influence the development of externalizing behaviors and internalizing behaviors in children and these foundations continue to affect behaviors in adulthood (4). The absence of a parental figure can present significant problems. As such, the vast majority of literature suggests that stark differences exist among left-behind children and non-left-behind children in terms of physical health and mental health outcomes. An extensive meta-analysis which aimed to analyse the effect of parental migration on the health of LBC in adolescents from low- and middle-income countries analyzed global data from more than 250,000 left-behind children (LBC) from 111 studies (1). They found that compared to non-left-behind children (NLBC), LBC had significantly increased risks for wasting, stunting, substance use, poorer psychological wellbeing, greater symptoms of depression, anxiety, suicidal ideation and conduct disorder (1). Of note, children exposed to parental migration had 1.7 times higher risk of experiencing suicidal ideation compared to controls (1).

Consistently ranked among the top five leading causes of death in children and adolescents, the World Health Organization describes suicide as a global health priority (5). Suicide is defined as a fatal self-injurious act with some evidence of intent to die (6). While preventable in its definition, annually, there are more than 700,000 reported cases of suicide around the world (7). Self-harm is defined as “an expression of personal distress by an individual who hurts him or herself” (8). An overlap between suicide and self-harm exists. Self-harm can be separated into two broad categories—non-suicidal self-injury, and self-injury with suicidal intent. According to the 5th edition of the Diagnostic and Statistical Manual of Mental Disorders, non-suicidal self-injury is defined as ‘self-injury directed to the surface of the body undertaken to induce relief from a negative feeling, and or cognitive state, or to achieve a positive mood state' (9). This article explores the broad term self-harm and does not distinguish between non-suicidal self-injury and self-injury with suicidal intent.

Regardless of whether self-harm is with or without suicidal ideation, self-harm may escalate into suicidal behaviors when it becomes insufficient as a coping strategy against trauma or stress (10), and individuals who self-harm may perceive a suicide attempt to be less frightening upon desensitization to pain (11). A recently published meta-analysis exploring prevalence of suicidal behaviors among LBC, found the prevalence of suicidal ideation was 18.7, 6.4% for suicide plan, and 3.1% for suicide attempt (12). LBC had 26% greater risk of having suicidal ideation than NLBC, and this was statistically significant (12).

Suicide behaviors among children are predictive for suicide behaviors in adulthood. One longitudinal study that prospectively traced the development of children from ages 5–30, examined whether suicidal ideation in community adolescents represents normative adolescent angst or is predictive of psychopathology, suicidal behaviors, and/or compromised functioning 15 years after onset (13). They found the risk of suicide attempts was increased by almost 12 folds in adolescents who described suicidal ideation at age 15, compared to those who denied of having suicidal ideations (13). Another study that aimed to investigate psychopathological consequences of University students who were LBC found the effects of parental migration perpetuate into adulthood as more of these University students reported suicide attempts (OR 1.67; 95%CI: 1.57–1.77, *p* < 0.001) and self-harm (OR 1.65; 95% CI: 1.53–1.79) than University students who were never ‘left-behind' (14).

Resilience is a multidimensional construct that can be explained as both a personality trait and a process (15). It is described as a characteristic relating to an adaptive stress resistant personal quality leading to healthier outcomes (16). Resilience is also defined as the dynamic process of overcoming the negative effects and trajectories associated with risk exposure and coping successfully with traumatic experiences (17). In addition to neural and psychological self-organizations, the transaction between the ecological context and the developing organism influences the resilience process (18).

The socioecological resilience framework proposes that the degree of positive emotional and behavioral development is determined by the child's interaction with their distal and proximal support systems including caregivers, family, school, peers, and the broader community (19). Successful support systems can promote positive feelings of security in individuals and their environment; whereas the availability of a child's primary caregiver in conjunction with their peers, school and broader community supports may promote healthy emotional and behavioral development in the context of parental absence as seen in LBC (1, 20). The socioecological resilience framework can also explain the significance of negative social factors, individual character, and negative psychological factors on unhealthy outcomes such as suicide (21, 22). Smoking and drinking alcohol have been described as negative coping of stressful life events when other supports are not available (23). A meta-analysis found smoking and alcohol use were risk factors for suicide ideation among mainland Chinese youth (24). Ultimately, the socioecological resilience framework emphasizes that individual traits, family aspects, and the social environment have a pivotal role in resilience (25).

Left-behind children are a unique population exposed to a lack of parental support and are thus at risk of development self-harm and suicidal ideation. No studies to date have used the socioecological resilience framework to describe correlates of suicidal ideation among left-behind children as important protective factors for healthy emotional and behavior development. Our research aims to fill in this gap by examining correlates of suicide among LBC including individual factors (negative coping, emotional and behavioral problems), duration of parental migration and negative school environment.

This project aims to (1) compare the prevalence of self-harm, suicidal ideation and psychological wellbeing among LBC and NLBC, and (2) to identify factors relating to suicidal ideation among LBC. We hypothesized that emotional and behavioral problems, negative coping behavior including smoking and drinking alcohol, poor school environment, were associated with suicidal ideation in left-behind children.

## Methodology

### Study design

This cross-sectional study collected data between November 2016 to January 2017 using purposive sampling. The reporting of the study followed the STROBE statement for observational study and had a rigorous quality control process. Firstly, all participating health professionals in the rural health centers were trained by United Nation's Child Fund assigned project experts. These trained health professionals went to each school, explained the study aim to each participating school teachers. All teachers contacted parents or caregivers of each student and obtained consent form from main caregivers. Students were also asked to provide consent before they understood the survey study. Schools were randomly selected to participate in the study. There is <2% missing data in this study as a result of the quality of training of people who conducted the data collection. Counties were recruited based on classification as “poverty-stricken area” defined as having two percent of the population living below the poverty line (per capita annual income of 2,300 Chinese Yuan ~360USD http://www.xinhuanet.com/english/2018-10/17/c_137538566.html). A total of 27 poverty-stricken counties were identified from 11 provinces and 1 municipality located in rural areas across China. In this study, left-behind children were defined as children registered in rural areas in Grade 1 of Junior school who had been exposed to at least 6 months of parental migration by either one parent or both parents. Non-left behind children were defined as children registered under a rural household who had not been exposed to parental migration. Grade 1 junior high school students were selected for two reasons—researchers were interested in exploring the students in their year of transition from primary schooler to high schooler, and grade 1 students would be most practical for follow-up if the study design was changed from cross-sectional to a longitudinal study.

### Participants

The sample size was calculated using the formula *N* = (*D*_*deffxZ*^2^*P*(1−*P*)/*d*^2^), with a confidence interval of 95% and z-value of 1.96. Probability *p* demonstrated the low prevalence of emotional and behavioral problems for LBC over 15.0%, and the design effect *D*_*deff*_ was 3 with a relative error of 15%; thus *d* = *15* × *15.0%*. Based on these calculations, the corresponding sample size was estimated to be ~2,900 with no <200 people from each province. The National Health and Family Planning Commission of PRC states research related to LBC should have a 2:1 ratio of LBC to NLBC (26). As per these recommendations, the study recruited 3,359 left-behind children and 1,667 non-left-behind children. The total sample size of 5,026 participants included is adequate for data analysis.

### Procedure

To ensure the population was adequately represented using post-weight adjustment, quota sampling was chosen to recruit participants. Grouping was performed based on reported number of LBC from each county. Further information was collected from each county including: (i) total number of children and their age, (ii) total number of LBC, (iii) ratio of boys to girls. Eleven provinces (Anhui, Guangxi, Guizhou, Hebei, Henan, Hubei, Hunan, Jiangxi, Shaanxi, Shanxi, Sichuan) and 1 municipality (Chongqing) which were identified to have a high proportion of left-behind children were selected. This study was part of an epidemiological survey on mental health of left-behind children. Its design was approved by local health administration bureaus, participating schools, and the Ethics Committee of Beijing Anding Hospital affiliated with Capital Medical University under ethical approval number of 2013 (06). The surveys on LBC and NLBC were collected from the same schools. Participation was voluntary and informed consent was obtained from both students and their caregivers. Students completed the survey in class under supervision from teachers who were briefed earlier by trained local health workers.

### Measures

#### Outcome measures: Self-harm and suicidality indicators

##### Self-harm

Assessment of self-harm was assessed based on the question—“In the past 6 months, have you intentionally hurt yourself (such as burn with a cigarette butt, cut with a blade, hit a wall with your head)?” The response was “yes” or “no”.

A self-assessment questionnaire asked students about markers of suicidality including suicidal ideation, suicide plan, previous suicide attempt and whether help was sought upon experiencing suicidal thoughts in the past 6 months. These were dichotomous variables responded with “yes” or “no”.

#### Risk factors for suicidality

According to resilience framework, risk factors were measured as individual level factors including emotional and behavior problems, socio-ecological factors including lack of caregiver support, peer bullying and negative school environment. Data in individual left-behind experience, and demographic characteristics were also collected.

##### Emotional and behavioral problem was measured by strengths and difficulties questionnaire

The Strengths and Difficulties questionnaire initially designed by Goodman (1997) is a globally used measurement tool for child and adolescent mental health and assessment of emotional and behavioral problems. The Chinese version of the SDQ is a reliable and valid instrument for measuring psychopathology in children and adolescents, as demonstrated by its satisfactory test-retest reliability, internal consistency, concurrent validity and discriminant validity (27, 28). The SDQ student edition contains 25 items assessing five subscales: (1) emotional symptoms; (2) conduct problem; (3) hyperactivity-inattention; (4) peer problems; and (5) prosocial behavior. Each item has three response options: “not true”, “somewhat true”, and “certainly true”, weighted with different scores of 0, 1 and 2 respectively; and reverse scoring for five items. The total scores for items under subscale 1 to 4 were combined to provide a total emotional and behavioral problem score, and subscale remained as an independent factor as prosocial behavior. The reliability for SDQ was 0.70 Cronbach's alpha and had reasonable level of reliability when it was applied to this new and unique population. Higher emotional and behavioral problems were associated with greater severity of psychological behavioral problems of the child.

##### School environment

The negative school environment score was derived from the combined score of 6 questions: (1) maliciously teased, (2) asked for property, (3) exclusion, (4) threatened, intimated, (5) physically harmed (hit, kicked), (6) teased due to appearance or other defect. Three response options were given: “never”, “occasionally”, “often”, weighted 0, 1, and 2 respectively. A high school environment score reflected poor school environment.

##### Negative psychological feeling of adolescents

Psychological feeling was attributed to the combined scores of 5 items: (1) unhappy because of stress or academic problems, (2) insomnia due to fear, (3) feelings of loneliness in past 6 months; (4) considered leaving home in past 6 months, (5) intentionally hurt yourself (burn with cigarette, cut blade). Those who described low psychological feeling were further asked if those feelings impacted: (1) family life, (2) relationship with friends, (3) study in class, (4) extracurricular activities, (5) burden others (family, friends, teachers). The difficulties impact score was calculated from the sum of these five items.

##### Demographic characteristics

Information of demographic characteristics were collected: gender, boarding status, frequency of outdoor activity, primary guardian, education of main guardian, history of engaging with cigarette smoking, history of engaging in drinking alcohol, duration of most recent paternal migration, duration of most recent maternal migration.

### Statistical analysis

Data was retrieved, coded and entered into Epidata 3.1 (Odense, Denmark). Categorical data such as demographic variables and self-harm were analyzed using chi-squared testing to identify potential confounding factors which may confound the relationship between suicidal ideation and independent variables. Independent samples *t*-test was used to compare continuous variables (emotional and behavioral problems, school environment, parenting questions, psychological characteristics) based on left-behind status and suicidality. Demographic variables found to significantly differ between adolescents who reported high suicidality and low suicidality were included in the subsequent multivariate analysis. Multiple logistic regression analysis assessed the association between school environment, adolescent psychological characteristics, emotional behavioral problems, self-harm with suicidality indicators in left-behind children. All statistical analyses were conducted by SPSS for Windows 28.0 (IBM, Chicago, IL) with statistical significance level defined as *p* < 0.05, two-tailed.

## Results

A total of 5,026 first year junior high school students-−3,359 (66.8%) LBC and 1667 (33.2%) NLBC—were included in the data analysis. All students who were invited to the study when data collection was conducted answered the survey. When 55 invalid surveys with more than 50% of questions were not answers and were excluded, this yielded high valid survey response rate of 98.9%. Table 1 illustrates a greater proportion of LBC stayed in dormitories (70.6%) compared to NLBC (62.6%; χ^2^ = 33.396, *p* < 0.001). The majority of LBC identified the primary caregiver to be their grandparents (88.9%), whereas among NLBC 94.1% reported their parents as the primary caregiver. More LBC (37.9%) reported having tried alcohol than NLBC (34.7%; χ^2^ = 8.045, *p* = 0.018). Significant differences were also found in the frequency of outdoor activity among LBC and NLBC (χ^2^ = 14.390, *p* = 0.002). There was no significant difference in gender (χ^2^ = 0.322, *p* = 0.570), previous smoking attempt (χ^2^ = 1.573, *p* = 0.210) or highest educational attainment of the primary caregiver (χ^2^ = 2.128, *p* = 0.712) based on parental migration experience.

**Table 1:** Comparison of demographic characteristics, smoking attempt, previous alcohol use in LBC (*n* = 3359) and NLBC (*n* = 1667).

**Variables**	**LBC n(%)**	**NLBC n(%)**	χ^2^	***p***	**Total n(%)**
**Sex**					
Male	1574 (46.9)	767 (46.0)	0.322	0.57	2341 (46.6)
Female	1785 (53.1)	900 (54.0)			2685 (53.4)
**Boarding status**					
In boarding school	2373 (70.6)	1043 (62.6)	33.396	<0.001	3416 (68.0)
Not in boarding school	986 (29.4)	624 (37.4)			1610 (32.0)
**Smoking attempt**					
Yes	483 (14.4)	218 (13.1)	1.573	0.21	701 (13.9)
Never smoked	2876 (85.6)	1449 (86.9)			4325 (86.1)
**Drinking attempt**					
Yes	1274 (37.9)	579 (34.7)	8.045	0.018	1853 (36.9)
Never drank alcohol	1993 (59.3)	1054 (63.2)			3047 (60.6)
**Outdoor activity**					
Everyday	1057 (31.5)	523 (31.5)	14.39	0.002	1580 (31.4)
Every week	926 (27.6)	527 (31.7)			1453 (28.9)
Every month	160 (4.8)	89 (5.4)			249 (5.0)
Rarely	1214 (36.2)	523 (31.5)			1737 (34.6)
**Identity of main caregiver**					
Father / Mother	47 (1.4)	1568 (94.1)	4389.89	<0.001	1615 (32.1)
Grandma/Grandpa	2987 (88.9)	84 (5.0)			3071 (61.1)
Other relatives (adults)	245 (7.3)	11 (4.3)			256 (5.1)
Non-adult relatives	80 (2.4)	3 (0.2)			83 (1.7)
**Caregiver highest education**					
Never went to school	808 (24.5)	25 (26.9)	2.128	0.712	34 (1.0)
Primary school	1564 (47.5)	45 (48.4)			169 (5.0)
Junior high school	724 (22.0)	17 (18.3)			741 (21.9)
Senior high school	165 (5.0)	4 (4.3)			1609 (47.5)
University	32 (1.0)	2 (2.2)			833 (24.6)
**Duration of previous father migration**					
<6 months	503 (15.0)				
6 months−1 year	1865 (55.7)				
1 year−2 year	762 (22.7)				
>2 years	213 (6.4)				
**Duration of previous mother migration**					
<6 months	643 (19.2)				
6 months−1 year	1980 (59.2)				
1 year−2 years	447 (13.4)				
>2 years	275 (8.2)				

As shown in Table 2, the overall prevalence of suicidal ideation among LBC was 7.2% which is significantly >5.5% reported by NLBC (χ^2^ = 4.854, *p* = 0.028). The prevalence of self-harm in the past 6 months was also significantly greater among LBC (16.4%) than NLBC (13.0%; χ^2^ = 10.232, *p* = 0.001).

**Table 2:** Prevalence of self-harm and suicidality indicators in LBC (*n* = 3344) and NLBC (*n* = 1663) using Chi-Square Analysis.

**Variables**	**LBC** ***n*****(%)**	**NLBC** ***n*****(%)**	χ^2^	***p***	**Total** ***n*****(%)**
**Suicidal ideation**					
Yes	240 (7.2)	92 (5.5)	4.854	0.028	332 (6.6)
No	3,104 (92.8)	1,571 (94.5)			4,675 (93.4)
**Self-harm in past 6 months**					
Yes	551 (16.4)	216 (13.0)	10.232	0.001	767 (15.3)
No	2,808 (83.6)	1451 (87.0)			4,259 (84.7)

Table 3 reports LBC experienced significantly poorer school environment (*M* = 8.812, SD = 1.927) than NLBC (*M* = 8.638, SD = 1.838; t-ratio=3.108, *p* < 0.001), lower psychological feeling (*M* = 9.427, SD = 2.911) than NLBC (*M* = 8.925, SD = 2.818; t-ratio=5.795, *p* < 0.001), and more emotional and behavioral problems (*M* = 13.089, SD = 5.303) than NLBC (*M* = 12.477, SD = 5.363, t-ratio=3.846, *p* < 0.001). There was no significant difference among LBC and NLBC based on prosocial behavior or how bothered the children were about their difficulties.

**Table 3:** Comparison of negative school environment, negative psychological feeling, emotional and behavior difficulties, bothered by difficulties of LBC and NLBC.

	**LBC mean (SD)**	**NLBC** **mean (SD)**	***t***	***p***	**Total mean (SD)**
1. Negative school environment	8.812 (1.927)	8.638 (1.838)	3.108	<0.001	8.754 (1.898)
2. Psychological feeling	9.427 (2.911)	8.925 (2.818)	5.795	<0.001	9.261 (2.888)
3. Bothered by difficulty	7.914 (2.386)	7.841 (2.335)	0.882	0.189	7.891 (2.368)
4. Emotional and behavioral problems	13.089 (5.303)	12.477 (5.363)	3.846	<0.001	12.891 (5.333)
5. Prosocial behavior	7.279 (1.945)	7.315 (2.011)	0.573	0.283	7.291 (1.966)

Table 4 suggests students who report suicidal ideation experienced more negative school environment, negative psychological wellbeing, more emotional and behavioral problems, and are more bothered by these difficulties compared to students who did not report suicidal ideation, and these all reached statistical significance of *p* < 0.001 among LBC and NLBC. For these four determinants, the magnitude of the *t*-value was consistently greater among LBC than NLBC. Only prosocial behavior was found not to have no relationship with suicidal ideation of students.

**Table 4:** Comparison of school environment, psychological feeling, total difficulties, prosocial behavior of LBC and NLBC by suicidality.

			**LBC (*****n** =* **3359)**				**NLBC (*****n** =* **1667)**	
	**Suicidal ideation mean (SD)**	**No suicidal ideation mean (SD)**	***t***	***p***	**Suicidal ideation mean (SD)**	**No suicidal ideation mean (SD)**	***t***	***p***
Negative school environment	10.408 (2.538)	8.689 (1.814)	10.291	<0.001	9.913 (2.256)	8.563 (1.784)	5.635	<0.001
Negative psychological feeling	12.871 (3.451)	9.161 (2.688)	16.274	<0.001	12.511 (3.317)	8.715 (2.640)	10.778	<0.001
Bothered by difficulty	9.473 (2.706)	7.753 (2.291)	8.693	<0.001	8.944 (2.951)	7.762 (2.266)	3.305	<0.001
Emotional behavioral problems	17.608 (5.873)	12.740 (5.093)	12.405	<0.001	16.703 (5.553)	12.228 (5.249)	7.878	<0.001
Prosocial behavior	7.129 (1.997)	7.291 (1.940)	−1.242	0.215	7.297 (2.068)	7.316 (2.008)	−0.087	0.931
	**LBC (*****n** =* **3359)**				**NLBC (*****n** =* **1667)**				**Total children**			
	**Suicide ideation mean (SD)**	**No suicidal ideation mean (SD)**	*t*	***p***	**Suicidal ideation mean (SD)**	**No suicidal ideation mean (SD)**	***t***	***p***	**Suicidal ideation mean (SD)**	**No suicidal ideation mean (SD)**	***t***	***p***
School environment	10.41 (2.54)	8.69 (1.81)	10.291	<0.001	9.91 (2.26)	8.56 (1.78)	5.635	<0.001	10.27 (2.47)	8.65 (1.80)	11.760	<0.001
Psychological feeling	12.87 (3.45)	9.16 (2.69)	16.274	<0.001	12.51 (3.32)	8.73 (2.64)	10.778	<0.001	12.77 (3.41)	9.01 (2.68)	19.644	<0.001
Bothered by difficulty	9.47 (2.71)	7.75 (2.29)	8.693	<0.001	8.94 (2.95)	7.76 (2.27)	3.305	<0.001	9.33 (2.78)	7.76 (2.28)	9.097	<0.001
Emotional behavioral problems	17.61 (5.87)	12.74 (5.09)	12.405	<0.001	16.70 (5.55)	12.23 (5.25)	7.878	<0.001	17.36 (5.79)	12.57 (5.15)	14.569	<0.001
Prosocial behavior	7.13 (2.00)	7.29 (1.94)	−1.242	0.215	7.30 (2.07)	7.32 (2.01)	−0.087	0.931	7.18 (2.01)	7.30 (1.96)	−1.108	0.268

According to Table 5, the greatest statistical difference observed among LBC who had suicidal ideation and those who denied of suicidal ideation was based on self-harm in the past 6 months. Among the LBC who had admitted to suicidal ideation, approximately half (55.0%) had performed self-harm in the past 6 months, while in those who denied of suicidal ideation only 13.4% had a history of self-harm (χ^2^ = 280.489, *p* < 0.001). A greater proportion of LBC who reported suicidal ideation had smoked in the past (29.2%) than those who denied of suicidal ideation (13.2%; χ^2^ =46.413, *p* < 0.001). Likewise, 61.9% of LBC with suicidal ideation reported having drunken alcohol before, compared to 37.3% who had drunken alcohol but never considered suicide (χ^2^ = 52.586, *p* < 0.001). In terms of demographic variables, suicidal ideation differed based on the highest educational attainment of the primary caregiver (χ^2^ = 22.478, *p* < 0.001). There was no significant difference in the distribution of student's gender, boarding status, frequency of outdoor activity, or the identify of primary caregiver based on suicidal ideation. Regarding NLBC, similar to LBC, there was significant difference (*p* < 0.001) in the proportion of students who had smoked or drunk alcohol in the past or performed self-harm in the past 6 months based on suicidal ideation. However, on the contrary, the proportion of NLBC who described suicidal ideation also differed based on student gender. NLBC of the female gender were more likely to report suicidal ideation than boys (χ^2^ = 7.032, *p* = 0.008).

**Table 5:** Suicidal ideation stratified by demographic characteristics, smoking attempt, previous alcohol use, and self-harm in LBC and NLBC.

	**Left-behind children**				**Non-left behind children**				**Total children**			
**Variables**	**SI (n%)**	**No SI n(%)**	χ^2^	***p***	**SI (n%)**	**No SI n(%)**	χ^2^	***p***	**SI (n%)**	**No SI n(%)**	χ^2^	***p***
**Sex**												
Male	100 (41.7)	1466 (47.2)	2.769	0.096	30 (32.6)	735 (46.8)	7.032	0.008	130 (39.2)	2201 (47.1)	7.822	0.005
Female	140 (58.3)	1638 (52.8)			62 (67.4)	836 (53.2)			202 (60.8)	2474 (52.9)		
**Boarding status**												
Yes	176 (73.3)	2189 (70.5)	0.85	0.356	60 (65.2)	981 (62.4)	0.285	0.593	236 (71.1)	3170 (67.8)	1.53	0.216
No	64 (26.7)	915 (29.5)			32 (34.8)	590 (37.6)			96 (28.9)	1505 (32.2)		
**Smoking attempt**												
Yes	70 (29.2)	409 (13.2)	46.413	<0.001	30 (32.6)	187 (11.9)	32.84	<0.001	100 (30.1)	596 (12.7)	78.163	<0.001
Never smoked	170 (70.8)	2695 (86.8)			62 (67.4)	1384 (88.1)			232 (69.9)	4079 (87.3)		
**Drinking attempt**												
Yes	139 (61.9)	1131 (37.3)	52.586	<0.001	54 (62.1)	523 (33.9)	28.533	<0.001	192 (61.9)	1654 (36.2)	81.86	<0.001
Never drank alcohol	85 (38.1)	1898 (62.7)			33 (37.9)	1019 (66.1)			118 (38.1)	2917 (63.8)		
**Self-harm**												
Yes	132 (55.0)	417 (13.4)	280.489	<0.001	43 (46.7)	173 (11.0)	98.159	<0.001	157 (47.3)	4085 (87.4)	384.901	<0.001
No	108 (45.0)	2687 (86.6)			49 (53.3)	1398 (89.0)			175 (52.7)	590 (12.6)		
**Outdoor activity**												
Everyday	62 (25.8)	990 (31.9)	5.096	0.167	23 (25.0)	499 (31.9)	2.49	0.477	85 (25.6)	1489 (31.9)	7.309	0.063
Every week	65 (27.1)	856 (27.6)			31 (33.7)	494 (31.5)			96 (28.9)	1350 (28.9)		
Every month	14 (5.8)	146 (4.7)			7 (7.6)	82 (5.2)			21 (6.3)	228 (4.9)		
Rarely	99 (41.3)	1110 (35.8)			31 (33.7)	491 (31.4)			1731 (34.6)	1601 (34.3)		
**Identity of main caregiver**												
Father/Mother	4 (1.7)	43 (1.4)	3.338	0.342	82 (89.1)	1484 (94.5)	6.602	0.086	86 (25.9)	1527 (32.7)	10.591	0.014
Grandma/Grandpa	205 (85.4)	2768 (89.2)			8 (8.7)	74 (4.7)			213 (64.2)	2842 (60.8)		
Other relatives	24 (10.0)	221 (7.1)			2 (2.2)	9 (0.6)			26 (7.8)	230 (4.9)		
Non adult relatives	7 (2.9)	72 (2.3)			0 (0.0)	3 (0.2)			7 (2.1)	75 (1.6)		
**Caregiver highest education**												
Never went to school	54 (23.4)	752 (24.7)	22.478	<0.001	5 (55.6)	19 (22.9)	7.577	0.108	59 (24.6)	771 (24.6)	19.745	<0.001
Primary school	109 (47.2)	1446 (47.5)			1 (11.1)	44 (53.0)			110 (45.8)	1490 (47.6) 686 (21.9)		
Junior high school	50 (21.6)	671 (22.0)			2 (22.2)	15 (18.1)			52 (21.7)	158 (5.0)		
Senior high school	9 (3.9)	155 (5.1)			1 (11.1)	3 (3.6)			10 (4.2)	25 (0.8)		
University	9 (3.9)	23 (0.8)			0 (0.0)	2 (2.4)			9 (3.8)			
**Duration of previous father migration**												
<6 months	36 (15.1)	469 (15.1)	3.439	0.329								
6 months – 1 year	134 (56.3)	1724 (55.6)										
1 year – 2 year	47 (19.7)	713 (23.0)										
>2 years	21 (8.8)	192 (6.2)										
**Duration of previous mother migration**												
<6 months	43 (18.0)	595 (19.2)	17.006	<0.001								
6 months – 1 year	136 (56.9)	1835 (59.4)										
1 year – 2 year	24 (10.0)	422 (13.7)										
>2 years	36 (15.1)	239 (7.7)										

Table 6 provides statistical evidence suggesting the relationship between negative school environment, negative psychological feeling, emotional and behavioral difficulties, self-harm, and past smoking attempt are significantly associated with suicidal ideation among LBC. The Nagelkerke variance of 30.2% indicates 30.2% of the variance in suicidal ideation can be explained by the psychosocial wellbeing markers, self-harm and the listed confounders.

**Table 6:** Correlates emotional and behavioral problems, smoking attempt, previous alcohol use and school environment on suicidal ideation in LBC.

**Variables**		**OR (95% CI)**	**p**
Poor school environment		1.109 (1.020–1.206)	0.015
Psychological feeling		1.242 (1.162–1.328)	<0.001
Bothered by difficulty		1.067 (0.993–1.147)	0.077
Emotional and behavioral problems		1.068 (1.031–1.108)	<0.001
Prosocial item		1.000 (0.912–1.096)	0.997
Self-harm in past 6 months	Yes	2.078 (1.394–3.100)	<0.001
	No	1	
Smoking	Yes	1.613 (1.053–2.473)	0.028
	No^a^	1	
Alcohol	Yes	1.311 (0.896–1.919)	0.163
	No^b^	1	
Duration of mother out to work	<6 months^c^	1	
	6 months-1yr	1.001 (0.611–1.641)	0.997
	1–2yr	0.729 (0.367–1.448)	0.367
	>2yrs	1.694 (0.897–3.200)	0.104
Education of main guardian	Never went to school^d^	1	
	Primary school	1.437 (0.917–2.250)	0.113
	Junior high school	1.400 (0.822–2.383)	0.216
	Senior high school	0.964 (0.378–2.459)	0.939
	University	3.756 (1.272–11.089)	0.017

*^a, b, c, d^ are reference groups. Nagelkerke variance explained by all independent variables are 30.2%*.

History of self-harm was the single greatest predictor for suicidal ideation among LBC. LBC who reported having performed self-harm in the past 6 months were 2.078 times more likely to report having suicidal ideation compared to those who had not self-harmed in the past 6 months (95% CI: 1.394–3.100, *p* < 0.001). Previous smoking attempt was the next significant risk factor; those who had smoked were 1.613 times more likely to report suicidal ideation (95% CI: 1.053–2.473, *p* = 0.028). Three out of the five psychosocial wellbeing determinants were found to be risk factors for suicidal ideation. One unit increase in psychological feeling score increased the chance that LBC experienced suicidal ideation by 24.2% (95% CI: 1.162–1.328, *p* < 0.001). Suicidal ideation was increased by 10.9% per unit for poor school environment (95% CI: 1.020–1.206, *p* = 0.015), and 6.8% for total difficulties (95% CI: 1.031–1.108, *p* < 0.001). In comparison, feeling bothered by difficulties (OR=1.067, *p* = 0.077), prosocial behavior (OR=1.000, *p* = 0.997), and past alcohol drinking attempt (OR=1.311, *p* = 0.163), and length of most recent maternal migration, were not found to be significantly associated with suicidal ideation among LBC.

## Discussion

Stemming from a socioecological resilience framework, our cross-sectional study examined correlates of suicidal ideation among LBC including emotional and behavioral problems, negative psychological feelings, negative school environmental in the absence of parental support, in conjunction with other unhealthy behaviors of smoking and drinking alcohol from a large representative LBC population drawn from impoverished areas of rural China. The overall prevalence of suicide ideation among left-behind was 7.2%, which was significantly different from 5.5% reported by controls. Our estimates for prevalence were markedly lower than the estimates of a recently published meta-analysis of 15 studies which reported the prevalence of suicide ideation among left-behind children as 18.7% (12). In comparing our results to Qu et al. (12), there are two possible explanations for this discrepancy. First, the majority of the studies included in their meta-analysis questions about suicidal behaviors in the last 12 months or in their lifetime, while in our study, the timeframe for self-harm and suicidal ideation was 6 months. Furthermore, some studies included provided scales for suicidality while we collected dichotomous data on self-harm and suicidal ideation.

We found 16.4% of left-behind children exhibited self-harm behaviors in the past 6 months, significantly different from the 13.0% reported by controls. Again, these estimates were substantially lower when compared to another population-based cross-sectional study of 2,898 children aimed to investigate self-harm behaviors and associated factors in LBC, and found 48% of LBC reported self-harm behaviors (29). In our study, self-harm was the single greatest risk factor for suicidal ideation among left-behind children (OR = 2.078, 95%CI: 1.394–3.100). Adolescents are particularly vulnerable to self-harm behaviors and affective disorders perhaps due to neurodevelopment involving cortical regions of the brain (30). The motives for self-harm are vast and variable including self-punishment, intrapersonal functions, expression of distress, and punishing others (31). Self-harm behaviors can transform into suicidal behaviors when the act of self-harm no longer serves as an effective coping method (10), and repeated acts of self-harm can desensitize individuals to pain (11). One meta-analyses that aimed to disengage the association of self-injurious thoughts and behaviors with subsequent suicidal behavior in adolescence and young adulthood, found a longitudinal association existed as suicide death was 22 times more likely (95%CI: 18.40–27.58) among adolescents and young adults who described any previous self-injurious thoughts or behaviors (32).

The significant higher proportion of adolescents experienced suicidal ideation can be explained by resilience model. Individual factors including emotion and behavior problems and psychological feeling ere significantly correlated with suicidality among LBC. Our findings are consistent with previous research which found a child's strengths and difficulties can predict self-harm (33) and suicidality (34). Our study suggests the relationship between smoking, alcohol and suicidal ideation may be the manifest of negative coping in response to stressful life events when other supports from parents, peers and community were absent (35).

According to socioecological resilience framework, when proximal support is lacking, the distal supports such as peer and school supports may compensate the missing support when parents were absent (19, 36), and reversely to double jeopardize adolescent's psychological development if school support is also lacking. Our study support the previous study's finding that poor school environment and support, particularly bullying victimization from peer, increased the likelihood of the risk outcome of suicidality in left-behind children (37). Experiences of being bullied had a significant relationship with suicide plan (38). In a study that examined the association between bullying victimization and suicidal ideation among adolescents, they found children who experienced both school bullying and cyberbullying victimization had 3.26 times higher odds (95%CI: 3.10–3.43) of experiencing suicidal ideation than controls (39). Bullying victimization is a negative life event that can be highly traumatic for individuals (40). Left-behind children who are victims of bullying had less self-compassion and hope, and greater feelings of depression, and these feelings are often associated with suicidal ideation (37), and consistent with our study finding.

Abundant evidence suggest that parental migration is a risk factor for suicidal ideation among left-behind adolescents (12, 14, 29, 41, 42). The innovation in this present study is this is the first study to implement the socioecological resilience framework to explain correlates of suicidality issues in LBC adolescent. We examined numerous factors such as individual factors (emotional and behavioral problems and psychological feeling,), negative school environment (peer support), and other negative coping behaviors of smoking and drinking alcohol. The new knowledge from the study is that coping behaviors, in particular, self-harm and smoking, as well as negative school environment and emotional and behavioral problems have multiply jeopardize left-behind adolescent's healthy psychological development which has further led to their suicidal ideation development. These findings ultimately emphasize the need to introduce substitute coping behaviors to assist left-behind children who are more vulnerable to stress and trauma than non-left-behind children. Future research should trial initiatives such as school-based suicide prevention programs, health-awareness programs, and mindfulness training to improve resilience, overall psychological wellbeing, and reduce suicidality of left-behind children.

In addition, we recruited participants based on sample representativeness targeting rural Chinese children living in areas of low socioeconomic status. We used nationwide data from 11 provinces and 1 municipal incorporating over 5,000 students which suggests generalisability of results to left-behind children living in impoverished areas of rural China.

Several limitations of this study must be acknowledged. First, the study design implemented was cross-sectional which means causal inferences cannot be established. Our data obtained 5 years ago may also present issues for application today due to revisions and reforms to policies surrounding left-behind children; nonetheless data on the impact of these changes LBC mental health cannot yet be ascertained. The study does not clearly distinguish self-harm from suicidal ideation as the broad definition for self-harm was used rather than asking participants specifically about non-suicidal self-injury. The potential overlap of the terms impacts on the integrity of the data, and thus results should be interpreted cautiously. While self-reported surveys are important tools to collect substantial amounts of data from many participants, the reliability of self-constructed school-based self-reported surveys may be questionable particularly due to fears for anonymity and the sensitivity of the survey asking about suicide. Many questions were retrospective in nature and recall bias could be introduced. Recruitment of participants by schools may also suggest selection bias as children who have dropped out were not included in the analysis.

Another limitation is that the study lacks generalisability to other year levels since participants were all year 1 junior high school students. Age has been reported as a predictor for adolescent suicidal ideation. One study which conducted multiple logistic regression to consider potential factors found all equations indicated risk of suicide attempts lowered as age increased (43). Finally, several key predictors such as depression, anxiety, stress, parent-child communication, for suicidal ideation were not investigated in this study. For example, one study found depression, anxiety, stress, hopelessness were the greatest risk factors of suicidal ideation in adolescents (44), while another study found healthy parent-child communication was a mediator for suicidal ideation among left-behind children (42). Future research should implement a longitudinal study design and incorporate depression and other mental health issues into the analysis to predict suicide behaviors. Stemming from a socioecological framework, further research may also explore the role of other support systems such as the government and extended family. These recommendations will help better understand the mechanism of how risk factors and protective factors influence suicide behaviors among a unique and vulnerable population of children.

## Conclusion

Findings in our study indicate suicidal ideation and self-harm were more prevalent among left-behind children than controls. As suggested by the socioecological resilience framework, our results support that adolescent's psychological characteristics including emotional and behavioral problems, negative psychological feeling, negative school environment, and negative coping behaviors (smoking, alcohol use, self-harm) are correlates of suicidal ideation in LBC. Self-harm was most positively associated with suicidal ideation in LBC. To prevent suicidal ideation and self-harm in left-behind children, there is urgent need for development of targeted strategies focusing on coping behaviors and emotion regulation, gatekeeper training of school teachers and peers, and psychosocial interventions for at-risk children.

## Data availability statement

The raw data supporting the conclusions of this article will be made available by the authors, without undue reservation.

## Ethics statement

The studies involving human participants were reviewed and approved by Ethics Committee of Beijing Anding Hospital affiliated with Capital Medical University under Ethical Approval Number of 2013 (06). Written informed consent to participate in this study was provided by the participants' legal guardian/next of kin.

## Author contributions

Y-mZ: collected data, conducted statistical analysis, drafted the manuscript, and edited and submitted the manuscript. LM: conducted statistical analysis, drafted the manuscript, and submitted the manuscript. C-xZ: organize the program, collected data, reviewed and revised the manuscript, and approved the final manuscript as submitted. FH: collected data, reviewed and revised the manuscript, and approved the final manuscript as submitted. X-nH and X-bT: reviewed and revised the manuscript, and approved the final manuscript as submitted. Y-z: conceptualized and designed the study, collected data, and approved the final manuscript as submitted. JS: designed the study, conducted statistical analysis, critically reviewed, edited and revised the manuscript, and approved the final manuscript as submitted. All authors contributed to the article and approved the submitted version.

## Funding

National “Twelfth Five-Year” Science and Technology Support Program (No: 2012BAI01B02); Research on prevention and control of major chronic non-communicable diseases in the Ministry of Science and Technology (No: 2016YFC1306100). Beijing Hospitals Authority Clinical medicine Development of special funding support, code: ZYLX202128.

## Conflict of interest

The authors declare that the research was conducted in the absence of any commercial or financial relationships that could be construed as a potential conflict of interest.

## Publisher's note

All claims expressed in this article are solely those of the authors and do not necessarily represent those of their affiliated organizations, or those of the publisher, the editors and the reviewers. Any product that may be evaluated in this article, or claim that may be made by its manufacturer, is not guaranteed or endorsed by the publisher.

## References

[1] Fellmeth G Rose-Clarke K Zhao C Busert LK Zheng Y Massazza A. Health impacts of parental migration on left-behind children and adolescents: a systematic review and meta-analysis. Lancet. (2018) 392:2567–82. 10.1016/S0140-6736(18)32558-3 30528471 PMC6294734

[2] UNICEF. Country Office Annual Report 2018 China. USA: UNICEF (2019).

[3] Pfeifer JH Berkman ET. The development of self and identity in adolescence: neural evidence and implications for a value-based choice perspective on motivated behavior. Child Dev Perspect. (2018) 12:158–64. 10.1111/cdep.12279 31363361 PMC6667174

[4] Hoskins DH. Consequences of parenting on adolescent outcomes. Societies. (2014) 4:506–31. 10.3390/soc4030506

[5] Qin J Albin B. The mental health of children left behind in rural China by migrating parents: a literature review. J Public Ment Health. (2010) 9:4–16. 10.5042/jpmh.2010.0458 (accessed December 23, 2021)21160544

[6] Turecki G Brent DA. Suicide and suicidal behaviour. Lancet. (2016) 387:1227–39. 10.1016/S0140-6736(15)00234-2 26385066 PMC5319859

[7] Ban L Guo S Scherpbier RW Wang X Zhou H Tata LJ. Child feeding and stunting prevalence in left-behind children: a descriptive analysis of data from a central and western Chinese population. Int J Public Health. (2017) 62:143–51. 10.1007/s00038-016-0844-6 (accessed December 20, 2021) 27318527 PMC5288445

[8] Wen M Lin D. Child development in rural China: Children left behind by their migrant parents and children of non-migrant families. Child development. (2012) 83:120–36. 10.1111/j.1467-8624.2011.01698.x 22181046

[9] American Psychiatric A American Psychiatric Association DSMTF editors. Diagnostic and statistical manual of mental disorders: DSM-5. Arlington, VA: American Psychiatric Association (2013).

[10] Whitlock J Knox KL. The relationship between self-injurious behavior and suicide in a young adult population. Arch Pediatr Adolesc Med. (2007) 161:634–40. 10.1001/archpedi.161.7.634 17606825

[11] Stewart SL Baiden P Theall-Honey L. Examining non-suicidal self-injury among adolescents with mental health needs, in Ontario, Canada. Arch Suicide Res. (2014) 18:392–409. 10.1080/13811118.2013.824838 24712902

[12] Qu G Shu L Zhang J Wu Y Ma S Han T. Suicide ideation, suicide plan, and suicide attempt among left-behind children and adolescents: a systematic review and meta-analysis. Suicide Life Threat Behav. (2021) 51:515–27. 10.1111/sltb.12731 33486779

[13] Reinherz HZ Tanner JL Berger SR Beardslee WR Fitzmaurice GM. Adolescent suicidal ideation as predictive of psychopathology, suicidal behavior, and compromised functioning at age 30. Am J Psychiatry. (2006) 163:1226–32. 10.1176/ajp.2006.163.7.1226 16816228

[14] Li X Coid JW Tang W Lv Q Zhang Y Yu H. Sustained effects of left-behind experience during childhood on mental health in Chinese University undergraduates. Eur Child Adolesc Psychiatry. (2020). 10.1007/s00787-020-01666-6 33113025

[15] Pooley JA Cohen L. Resilience: a definition in context. Aust Commun Psychol. (2010) 22:30–7. 10.1192/bjp.170.5.447 9307695

[16] Ahern NR Ark P Byers J. Resilience and coping strategies in adolescents–additional content. Nurs Child Young People. (2008) 20:32–6. 10.7748/paed2008.12.20.10.1.c6905 19119747

[17] Fergus S Zimmerman MA. Adolescent resilience: a framework for understanding healthy development in the face of risk. Annu Rev Public Health. (2005) 26:399–419. 10.1146/annurev.publhealth.26.021304.144357 15760295

[18] Curtis WJ Cicchetti D. Emotion and resilience: a multilevel investigation of hemispheric electroencephalogram asymmetry and emotion regulation in maltreated and nonmaltreated children. Dev Psychopathol. (2007) 19:811–40. 10.1017/S0954579407000405 17705904

[19] Zhou YM Zhao CX Qi YJ Fan H Huang XN Tian XB. Emotional and behavioral problems of left-behind children in impoverished rural china: a comparative cross-sectional study of fourth-grade children. J Adolesc Health. (2020) 67:S48–54. 10.1016/j.jadohealth.2020.06.016 33246533

[20] Yang R Zhang L Wu X Fu Q Bao Q. Caregivers' mind-mindedness and rural left-behind young children's insecure attachment: the moderated mediation model of theory of mind and family status. Child Abuse Negl. (2022) 124:105472. 10.1016/j.chiabu.2021.105472 34991010

[21] Zolkoski SM Bullock LM. Resilience in children and youth: a review. Child Youth Serv Rev. (2012) 34:2295–303. 10.1016/j.childyouth.2012.08.009

[22] Garmezy N Masten AS Tellegen A. The study of stress and competence in children: a building block for developmental psychopathology. Child Dev. (1984) 55:97–111. 10.2307/1129837 6705637

[23] Pompili M Serafini G Innamorati M Dominici G Ferracuti S Kotzalidis GD. Suicidal behavior and alcohol abuse. Int J Environ Res Public Health. (2010) 7:1392–431. 10.3390/ijerph7041392 20617037 PMC2872355

[24] Li Y Li Y Cao J. Factors associated with suicidal behaviors in mainland China: a meta-analysis. BMC Public Health. (2012) 12:1–13. 10.1186/1471-2458-12-524 22800121 PMC3490836

[25] Ungar M. Resilience across cultures. Br J Social Work. (2008) 38:218–35. 10.1093/bjsw/bcl343

[26] Tang D Choi WI Deng L Bian Y Hu H. Health status of children left behind in rural areas of Sichuan Province of China: a cross-sectional study. BMC Int Health Hum Rights. (2019) 19:4. 10.1186/s12914-019-0191-9 30691456 PMC6350297

[27] Liu S-K Chien Y-L Shang C-Y Lin C-H Liu Y-C Gau SS-F. Psychometric properties of the Chinese version of strength and difficulties questionnaire. Compr Psychiatry. (2013) 54:720–30. 10.1016/j.comppsych.2013.01.002 23433222

[28] Yao S Zhang C Zhu X Jing X McWhinnie CM Abela JR. Measuring adolescent psychopathology: psychometric properties of the self-report strengths and difficulties questionnaire in a sample of Chinese adolescents. J Adolesc Health. (2009) 45:55–62. 10.1016/j.jadohealth.2008.11.006 19541250

[29] Xiao YY He LP Chang W Zhang SN Wang R Chen XW. Self-harm behaviors, suicidal ideation, and associated factors among rural left-behind children in west China. Ann Epidemiol. (2020) 42:42–9. 10.1016/j.annepidem.2019.12.014 31992492

[30] Hawton K Saunders KE O'Connor RC. Self-harm and suicide in adolescents. Lancet. (2012) 379:2373–82. 10.1016/S0140-6736(12)60322-5 22726518

[31] Taylor PJ Jomar K Dhingra K Forrester R Shahmalak U Dickson JM. meta-analysis of the prevalence of different functions of non-suicidal self-injury. J Affect Disord. (2018) 227:759–69. 10.1016/j.jad.2017.11.073 29689691

[32] Castellvi P Lucas-Romero E Miranda-Mendizabal A Pares-Badell O Almenara J Alonso I. Longitudinal association between self-injurious thoughts and behaviors and suicidal behavior in adolescents and young adults: a systematic review with meta-analysis. J Affect Disord. (2017) 215:37–48. 10.1016/j.jad.2017.03.035 28315579

[33] Cassels M Baetens I Wilkinson P Hoppenbrouwers K Wiersema JR Van Leeuwen K. Attachment and non-suicidal self-injury among young adolescents: the indirect role of behavioral problems. Arch Suicide Res. (2019) 23:688–96. 10.1080/13811118.2018.1494651 30118634

[34] Rodriguez-Blanco L de Neira MD Garcia-Nieto R Zamorano-Ibarra MJ Ramos-Garcia S Segura-Frontelo A. Victimization exposure and suicidal ideation among Spaniard adolescents evaluated at outpatient mental health services. Int J Adolesc Med Health. (2015) 27:213–9. 10.1515/ijamh-2015-5014 25389986

[35] Bobo JK Husten C. Sociocultural influences on smoking and drinking. Alcohol Res Health. (2000) 24:225–32. 15986717 PMC6709745

[36] Sun J Stewart D. Development of population-based resilience measures in the primary school setting. Health Educ. (2007). 10.1108/0965428071082795734398201

[37] Zhang H Chi P Long H Ren X. Bullying victimization and depression among left-behind children in rural China: Roles of self-compassion and hope. Child Abuse Negl. (2019) 96:104072. 10.1016/j.chiabu.2019.104072 31319239

[38] Bullying Victimization S. and Suicide ideation and plan: focusing on youth in low- and middle-income countries. J Adolesc Health. (2020) 66:115–22. 10.1016/j.jadohealth.2019.07.006 31515133

[39] Baiden P Tadeo SK. Investigating the association between bullying victimization and suicidal ideation among adolescents: evidence from the 2017 Youth Risk Behavior Survey. Child Abuse Negl. (2020) 102:104417. 10.1016/j.chiabu.2020.104417 32113078

[40] Nielsen MB Tangen T Idsoe T Matthiesen SB Magerøy N. Post-traumatic stress disorder as a consequence of bullying at work and at school. A literature review and meta-analysis. Aggression Violent Behav. (2015) 21:17–24. 10.1016/j.avb.2015.01.001

[41] Tian X Chang W Meng Q Chen Y Yu Z He L. Resilience and self-harm among left-behind children in Yunnan, China: a community-based survey. BMC Public Health. (2019) 19:1728. 10.1186/s12889-019-8075-4 31870359 PMC6929398

[42] Lu J Lin L Roy B Riley C Wang E Wang K. The impacts of parent-child communication on left-behind children's mental health and suicidal ideation: a cross sectional study in Anhui. Child Youth Serv Rev. (2020) 110:104785. 10.1016/j.childyouth.2020.104785 32801409 PMC7425803

[43] Chang HJ Yan QG Tang L Huang J Ma YQ Ye XZ. A comparative analysis of suicide attempts in left-behind children and non-left-behind children in rural China. PLoS ONE. (2017) 12:15. 10.1371/journal.pone.0178743 28594874 PMC5464573

[44] Primananda M Keliat BA. Risk and Protective Factors of Suicidal Ideation in Adolescents. Compr Child Adolesc Nurs. (2019) 42:179–88. 10.1080/24694193.2019.1578439 31192739

